# The Impact of COVID-19 on Tuberculosis Program Performance in the Kingdom of Lesotho

**DOI:** 10.3390/tropicalmed8030165

**Published:** 2023-03-11

**Authors:** Afom T. Andom, Donald Fejfar, Courtney M. Yuen, Melino Ndayizigiye, Jean Claude Mugunga, Joia S. Mukherjee

**Affiliations:** 1 Partners in Health-Lesotho, Maseru 0100, Lesotho; 2 Partners in Health, Boston, MA 02199, USA; 3 Department of Global Health and Social Medicine, Harvard Medical School, Boston, MA 02115, USA; 4 Division of Global Health Equity, Brigham and Women’s Hospital, Boston, MA 02115, USA

**Keywords:** COVID-19, tuberculosis, essential health services, time series modeling, health policy

## Abstract

Background: As tuberculosis (TB) is an airborne disease requiring multi-month therapy, systems of TB detection and care were profoundly impacted by the COVID-19 pandemic. The worsening economic situation, including income, food, and housing insecurity, impacted the social conditions in which TB—already a leading killer in resource-limited settings—thrives. This study assesses the impact of COVID-19 on TB detection and treatment in Lesotho. Methods: We used routine program data from 78 health facilities in Lesotho. We created time series models from July 2018 to March 2021 to quantify COVID-19-related disruptions to TB program indicators: outpatient visits; presumptive, diagnosed, treated, and HIV co-infected cases; and treatment outcomes including successful (cured and completed) and unsuccessful (death and treatment outcome unknown). Results: We observed a significant decline in cumulative outpatient visits (−37.4%, 95% prediction interval [PI]: −40.1%, −28.7%) and new TB cases diagnosed (−38.7%, 95%PI: −47.2%, −28.4%) during the pandemic, as well as TB-HIV co-infections (−67.0%, 95%PI: −72.6%, −60.0%). However, we observed no difference in treatment success (−2.1%, 95%PI: −17.0%, 15.8%). Conclusions: TB case detection in Lesotho fell during the COVID-19 pandemic, likely related to the uptake of overall health services. However, treatment success rates did not change, indicating a strong health system and the success of local strategies to maintain treatment programs.

## 1. Introduction

Tuberculosis (TB) remains a major public health threat globally [1]. It disproportionally affects people with low socioeconomic status [1,2]. As a result, the major burden of TB has been in lower and middle-income countries [2]. COVID-19 has exacted a direct death toll on millions of people. But millions of people have fallen ill and died due to both the disruption in health services and the deterioration of economic status of the population [3]. Both of these indirect impacts of COVID-19 will substantially increase the number of individuals at a greater risk of acquiring TB and receiving adequate detection and care [4].

Worldwide, rapid spread of COVID-19 globally has significantly reversed the milestones and ambitious targets of the TB program [5,6,7,8]. Governments imposed various public health measures such as travel restrictions, curfews, local and international transport bans, and school closures to contain the spread of COVID-19. The measures taken to curb the COVID-19 pandemic significantly hamper access to TB services, particularly in developing countries where the quality of TB measures was sub-optimal even before the start of COVID-19 [9,10]. Additionally, many governments—particularly in developing countries—are forced to divert resources from the ambitious target of TB control to tackle the spread of COVID-19 [11,12,13]. The synergy of both low socioeconomic status and COVID-19 significantly jeopardize the important milestones registered in the TB program [14]. Africa roughly contributed 24% of the TB burden globally [1]. The burden of TB remains as a significant threat, particularly in countries with high HIV burden such as Lesotho.

The health system of Lesotho has been significantly overburdened with a high prevalence of HIV and TB diseases. Lesotho is the country with the highest TB incidence at 614 cases per 100,000 population [1] and the second highest in HIV prevalence in the world at 22.7% [15]. The HIV coinfection rate among TB patients was 55% [1].

With the rapid spread of COVID-19 in May 2020, the health system of Lesotho was challenged to simultaneously respond to the rapid spread of COVID-19 and to sustain the ambitious target to improve TB case detection and treatment. Even prior to the pandemic, only 50% of expected TB cases were detected annually in Lesotho. That dismal figure fell to 32% in the 2022 WHO report [1]. Understanding the effect of COVID-19 on TB services is urgently needed to design rapid mitigation strategies to address the dire situation of TB Lesotho. This study aims to assess the magnitude of COVID-19-related disruption in the TB care cascade including care seeking, diagnosis, treatment initiation, and treatment completion of TB in Lesotho.

## 2. Materials and Methods

### 2.1. Study Setting

Lesotho is a small and mountainous country entirely within the borders of South Africa. It has a population of 2.2 million people, an HIV prevalence of 22% [15], and a TB incidence of 614 per 100,000 [1]. Lesotho comprises 10 administrative districts [16]. Lesotho’s health services are delivered at three levels, namely primary, secondary, and tertiary levels. There were 286 public health facilities in Lesotho in 2016, including 265 primary health care centers, 20 general district hospitals, and a multi-specialty tertiary hospital (Queen Mamohato Memorial Hospital) located in Maseru. In addition, a network of more than 6000 village health workers provides basic health services at a community level [17].

Laboratory diagnostic and imaging services such as X-ray, sputum smear microscopy and Xpert MTB/RIF testing, chemistry, and full blood count are mainly available at district hospitals. The majority of health centers have to send samples to the district hospital labs for processing of samples. Additionally, the majority of health centers do not have X-ray machines and those that are available are analogue and mainly available in the district hospitals. There is one National Reference Laboratory in the capital, Maseru, that provides specialized laboratory services including TB diagnostic services such as culture and drug susceptibility testing [17]. Prior to the pandemic, TB treatment was normally dispensed for two weeks at the initial visit, followed by monthly refills. During the pandemic, the Ministry of Health adopted a strategy of multi-month dispensing of TB treatment to stable patients to provide uninterrupted and timely TB treatment.

The health care distribution in the country follows a typical pattern with 10–15 primary care clinics referring to one of 10 public and public–private district hospitals. The rural highlands are very remote, often without proper roads and impassible during snow or rain. While the diagnosis of TB—by GeneXpert test on sputum—is free, the tests are only performed at district hospitals. This situation requires specimens to be transported—a process often marked by significant delays. Radiologic diagnosis is not free and can only be performed at district hospitals, requiring the long-distance travel of patients.

The study was conducted in 78 health facilities of Lesotho in which PIH Lesotho works, not including the only tertiary care center in Lesotho, but including seven district hospitals and 71 primary care centers. The health facilities serve around half of Lesotho’s population.

### 2.2. Study Population

The study population included all individuals who visited the 78 health facilities during July 2018 to March 2021 either through the outpatient department or as patients with TB. This includes both adults and children.

### 2.3. Data Collection

We selected seven indicators (Table 1) because of their clinical importance to the TB program, the availability of data in the routine health system, and the high quality of data across all health facilities and months (from July 2018 to March 2021). The selected indicators are number of outpatient department visits, number of presumptive TB cases, number of diagnosed TB cases, number of cases of TB/HIV coinfection, percentage of patients with successful TB treatment (people who completed TB treatment or were cured of TB), percentage of TB patients who died while on TB treatment, and percentage of TB patients whose treatment outcome was unknown (a category which includes people lost from the program). Data was pulled from an internal PIH database tracking the TB program. Monthly aggregate count data were collected from each site; data were not disaggregated by age, sex, or other individual characteristics.

### 2.4. Analysis

We conducted a time series analysis of aggregate data. We modeled monthly outcomes as counts (outpatient visits; presumptive, diagnosed, treated, and HIV co-infected cases) or percentages (treatment success and died, unknown treatment outcomes) at the facility level based on yearly trends and seasonality using baseline data from July 2018 to February 2020. This was done using the following generalized linear model with negative binomial distribution and log-link, where *Y* indicates the monthly indicator count, *t* indicates the cumulative month number, and *K* indicates the number of harmonic functions to include (we take *K* to be 3). The year term captures a long-term annual trend, and the harmonic terms capture seasonality:

$$\log(E[Y\;|\;year,\;t])=\beta_{0}+\beta_{1}year_{t}+\sum_{k=1}^{K}\beta_{3k}\cos(\frac{2\pi kt}{12})+\beta_{4k}\sin(\frac{2\pi kt}{12})$$

We used these models to extrapolate predicted values for each indicator from March 2020 to March 2021. This was considered the pandemic era, since both national pandemic responses and cases began to circulate in the region in March 2020 and other studies began to observe drops in health service utilization at this time [18]. Predicted values were aggregated across all sites to provide estimates representing all health facilities in the analysis, which is what is reported here. Deviations from the observed values are reported as proportion deviations with 95% prediction intervals (PIs) in time series graphs, as well as cumulative estimated deviations and estimated proportion deviations from the observed values of the entire pandemic era along with their respective 95% PIs in a table. A significant result represents an estimate and 95% PI that lies entirely above or below zero. R and RStudio (v4.0.4) were used to clean, analyze, and visualize the data.

## 3. Results

**Figure 1:**
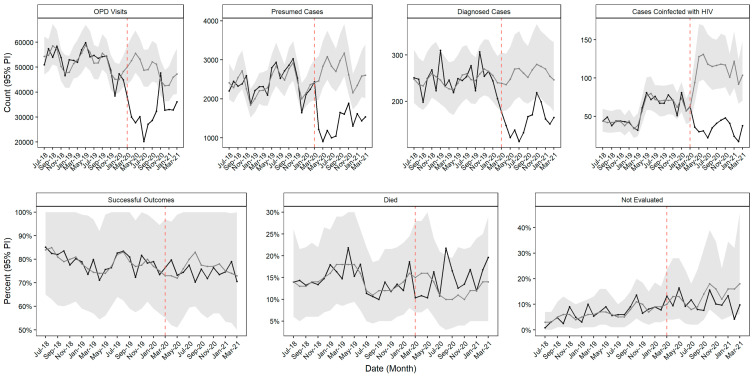
Time series models of seven TB programs, by month and indicator, July 2018 to March 2021. Grey lines show expected values and grey shading indicates 95% prediction intervals; black lines show observed values.

In the baseline period, there were an average of 53,748 (interquartile range [IQR] 50,455, 54,482) outpatient department visits, 2357 (IQR 2209, 2626) presumptive TB cases, and 249 (IQR 230, 257) TB cases across the 78 health facilities each month. During this period, an average of 79.5% (IQR 76.2%, 82.1%) patients had successful TB treatment outcome each month. We observed significant drops in the number of outpatient visits and presumed, diagnosed, and HIV-co-infected TB cases throughout the pandemic era (Figure 1, Table 2). The number of outpatient visits was below expected for all months except November 2020, with a cumulative deviation from expected of −34.7% (95% PI −40.1%, −28.7%). The number of presumed cases was as expected in March 2020 but fell for the rest of the pandemic era, with a cumulative deviation from the expected of −46.1% (95% PI −52.2%, −39.0%). The number of diagnosed cases was below expected in all months except November 2020, with a cumulative deviation from expected of −38.7% (95% PI −47.2%, −28.4%). Similar to presumed cases, the number of TB cases co-infected with HIV was as expected in March 2020, but fell thereafter. The cumulative deviation from expected was −67.0% (95% PI −72.6%, −60.0%).

The percentage of total 12-month outcomes that were successful (cured or completed treatment), death, or not evaluated remained as expected throughout the majority of the pandemic era, except for a slightly above-expected proportion of deaths in August 2020 and a slightly below the expected proportion of not evaluated outcomes in February 2021 (Figure 1, Table 2). The cumulative proportion deviation was not significantly different from expected for any 12-month outcome.

## 4. Discussion

In our study, we observed a significant drop in outpatient department visits and this likely contributed to the low number of presumptive and diagnosed TB cases. However, TB treatment outcomes did not change significantly. The decline in outpatient department visits is likely due to the COVID-19 lockdown, movement restrictions, and restriction of public transport that would hinder the opportunities for those experiencing TB symptoms to seek care [19]. People with TB also might not have come to the health facilities to get services due to fear of contracting COVID-19 [20]. Even when people with TB were able to visit the health facilities, they might not have received adequate TB services due to a lack of diagnostics and a shortage of manpower [21]. However, once TB patients started treatment, treatment outcomes remained unaffected, reflecting the success of a strategy employed by the Ministry of Health to provide uninterrupted and timely TB treatment services through multi-month dispensing of TB treatment to stable patients, as well as a well-structured and compensated network of village health workers. Village health workers may have played a significant role in the home-based follow-up, monitoring, and accompaniment of TB patients to health facilities.

Similar to our study, various studies from other countries have shown an overall decrease in TB diagnoses and treatment initiations. Studies conducted in 2020 in China and Nigeria, and in Malawi, Kenya, Sierra Leone and Zimbabwe in 2021, demonstrated that during the pandemic there was an overall decrease in people with presumptive TB, TB diagnosis, and treatment initiation [21,22,23,24,25,26]. Common reasons for reduced usage of TB services across settings include fear of getting infected with COVID-19, transport disruptions, and movement restrictions [27].

There has been variation across countries in the impact of the pandemic on TB treatment outcomes. A study from Sierra Leone found improved treatment success during the pandemic [23], while a study from Zimbabwe found decreased treatment success [24]. Studies from China, Nigeria, Malawi, and Kenya found no change in treatment success [21,22,23,24], similar to our findings. In countries that sustained or improved TB treatment success, different approaches were used to promote the continuity of TB services such as introducing task shifting to bridge the gaps of shortage of human resources, improved community outreaches to provide information on service disruption, and introduction of a multi-month drug dispensary system [28].

The studies in Malawi and Kenya observed a decrease in HIV testing during the COVID-19 pandemic with the increased implementation of HIV self-testing [23,24]. It is unclear whether increased self-testing contributed to our observation that the number of diagnosed HIV/TB cases dropped more than the number of cases of TB diagnosed during the COVID-19 pandemic. Self-testing relies on patients with a positive test result coming to the health facilities for HIV confirmatory tests and linkage to care; if patients who test positive at home do not come to the health facility, they cannot be screened for TB, which could contribute to the reduced detection of TB/HIV coinfection.

The study has some limitations. To begin with, our study used data aggregated at the monthly and facility level, which precluded disaggregation by potentially important patient characteristics such as age or gender, and we were not able to provide the demographic characteristics of the study population. However, we tried to include data that can represent the whole country and different geographical distributions. Additionally, we could not explore causes of change in TB service usage or diagnoses, only inferring them based on the timing of the pandemic. Furthermore, we have no direct data on service provision in health facilities, therefore, we could not evaluate the impact of specific changes in service delivery.

## 5. Conclusions

The COVID-19 pandemic impacted TB case detection in Lesotho, likely by reducing overall usage of health services. However, efforts to ensure the continuity of TB treatment services were sufficient to maintain treatment success rates at pre-pandemic levels. Building a resilient health system that can tolerate any epidemic is critically important for the continuity of health services. Strengthening all components of health systems equally, such as health service delivery, the health workforce, health information systems, access to essential medicines and medical products, and health system financing, leadership, and governance are critically important for the sustainability and continuity of health services including TB programs. Systems thinking is important to identify gaps in health service delivery. An interconnected and structured health system is vital for the success of the TB epidemic.

## Figures and Tables

**Table 1:** Selected indicator definitions.

Indicators	Definitions
Outpatient department (OPD) visits	Visits consulted at the outpatient department in facilities excludes patients for antenatal care visit, deliveries, postnatal care visit, tuberculosis, anteretroviral therapy, pediatrics (under 5), HIV testing, and other clients not seen for acute or chronic illness.
Presumed TB cases	A patient who presents with symptoms or signs suggestive of TB.
Diagnosed TB cases	Any bacteriologically confirmed or clinically diagnosed case of TB.
Cases of TB/HIV co-infection	Any person with a bacteriologically confirmed or clinically diagnosed case of TB who has a positive result from HIV testing conducted at the time of TB diagnosis or other documented evidence of enrolment in HIV care.
Percentage of patients with successful TB treatment outcomes	The number of TB patients who successfully completed TB treatment or were cured of TB disease out of the number of TB patients who started treatment.
Percentage of TB patients who died during treatment	The number of TB patients who died for any reason during the course of TB treatment out of these patients who started treatment.
Percentage of TB patients with unknown treatment outcomes	The number of TB patients for whom no treatment outcome was assigned out of the patients who started TB treatment. This includes cases transferred out to another treatment unit as well as cases for whom the treatment outcome is unknown to the reporting unit.

**Table 2:** Cumulative deviations in seven TB program indicators during March 2020 to March 2021 (pandemic period) compared to trends established during July 2018–February 2020 (baseline period).

Indicator	Monthly Value During Baseline, Median (IQR)	Cumulative Predicated Value during Pandemic, Median (IQR)	Cumulative Observed Value during Pandemic	Cumulative Deviation from Expected, 95% PI	Cumulative Proportion Deviation from Expected, 95% PI
Outpatient department visits	53,748 (50,455, 54,482)	638,178 (584,386, 695,632)	416,445	−221,733.0 (−279,186.7, −167,940.9)	−34.7% (−40.1%, −28.7%)
Presumed TB cases	2357 (2209, 2626)	34,947 (30,892, 39,422)	18,837	−16,109.5 (−20,585.4, −12,054.5)	−46.1% (−52.2%, −39.0%)
Diagnosed TB cases	249 (230, 257)	3367 (2881, 3909)	2064	−1303.0 (−1844.8, −817.3)	−38.7% (−47.2%, −28.4%)
Cases of TB/HIV co-infection	54 (44, 71)	1427 (1177, 1721)	471	−955.5 (−1250.2, −705.5)	−67.0% (−72.6%, −60.0%)
Percentage of patients with successful treatment outcomes	80 (76, 82)	995 (841, 1174)	974	−21.0 (−200.0, 132.5)	−2.1% (−17.0%, 15.8%)
Percentage of TB patients who died during treatment	14 (13, 16)	170 (112, 245)	188	18.2 (−56.8, 76.7)	10.7% (−23.2%, 68.8%)
Percentage of TB patients with unknown treatment outcomes	6 (5, 9)	177 (101, 310)	138	−38.7 (−171.7, 37.3)	−21.9% (−55.4%, 36.9%)

## Data Availability

Due to data privacy concerns, data is not made publicly available. However, reasonable data requests may be granted through contacting the corresponding author.
